# MicroRNAs and Potential Targets in Osteosarcoma: Review

**DOI:** 10.3389/fped.2015.00069

**Published:** 2015-08-24

**Authors:** Valerie B. Sampson, Soonmoon Yoo, Asmita Kumar, Nancy S. Vetter, E. Anders Kolb

**Affiliations:** ^1^Nemours Center for Cancer and Blood Disorders, Alfred I. duPont Hospital for Children, Wilmington, DE, USA; ^2^Nemours Biomedical Research, Alfred I. duPont Hospital for Children, Wilmington, DE, USA

**Keywords:** osteosarcoma, microRNA, mutations, cell proliferation, apoptosis

## Abstract

Osteosarcoma is the most common bone cancer in children and young adults. Surgery and multi-agent chemotherapy are the standard treatment regimens for this disease. New therapies are being investigated to improve overall survival in patients. Molecular targets that actively modulate cell processes, such as cell-cycle control, cell proliferation, metabolism, and apoptosis, have been studied, but it remains a challenge to develop novel, effective-targeted therapies to treat this heterogeneous and complex disease. MicroRNAs (miRNAs) are small non-coding RNAs that play critical roles in regulating cell processes including growth, development, and disease. miRNAs function as oncogenes or tumor suppressors to regulate gene and protein expression. Several studies have demonstrated the involvement of miRNAs in the pathogenesis of osteosarcoma with the potential for development in disease diagnostics and therapeutics. In this review, we discuss the current knowledge on the role of miRNAs and their target genes and evaluate their potential use as therapeutic agents in osteosarcoma. We also summarize the efficacy of inhibition of oncogenic miRNAs or expression of tumor suppressor miRNAs in preclinical models of osteosarcoma. Recent progress on systemic delivery as well as current applications for miRNAs as therapeutic agents has seen the advancement of miR-34a in clinical trials for adult patients with non-resectable primary liver cancer or metastatic cancer with liver involvement. We suggest a global approach to the understanding of the pathogenesis of osteosarcoma may identify candidate miRNAs as promising biomarkers for this rare disease.

## Introduction

Osteosarcoma (OS) is an aggressive bone cancer that affects children and adolescents. Approximately 60% of cases are pediatric patients between 10 and 20 years of age (1). Several studies suggest that OS arises from primitive mesenchymal bone-forming cells that undergo aberrant alterations in the differentiation program. This results in a heterogenic cancer, with complex etiology, characterized by vast genomic instability, highly abnormal karyotypes, and multiple genomic aberrations with copy number gains and losses occurring at multiple chromosomes (2, 3). Patients with certain rare and inherited syndromes, such as Li–Fraumeni syndrome, hereditary retinoblastoma, Rothmund–Thomson syndrome, Bloom syndrome, and Werner syndrome have a higher incidence of OS (4). Treatment involves standard chemotherapy administered before and after surgery, and may be followed by radiation, which achieves a 5-year survival rate of 60–70% of patients. However, the survival of patients with locally advanced or metastatic tumors at diagnosis, and recurrent disease remains low (~20%), and the median survival time for these patients is only 23 months (5). Current clinical trials of cytotoxic chemotherapy and targeted agents may achieve an objective response in a subset of patients with OS, but have not increased overall survival in the recent treatment era. Further, constitutive and acquired resistance to these therapies remains a clinical challenge. Therefore, a global understanding of the underlying factors of tumor biology will assist in the identification of diagnostic and prognostic markers and therapeutic targets for the management of patients with OS.

MicroRNAs (miRNAs) belong to the group of small, non-coding, regulatory RNA molecules, ranging between 18 and 25 nucleotides in length (6). They recognize and bind specific target mRNAs by complete or partial base-pairing mostly at the 3′-untranslated region (UTR) of the target genes to post-transcriptionally regulate gene expression. Since their discovery nearly 20 years ago, bioinformatics and biological studies have identified >1,000 miRNAs that regulate possibly 50% of human genes. Each miRNA likely controls hundreds of gene transcripts (7). The sequences of miRNAs are highly conserved among distantly related organisms, indicating their participation in essential biological processes as development, cellular differentiation, metabolism, proliferation, and apoptosis. Moreover, they regulate biological systems as stemness, immunity, and cancer. Studies show that more than 50% of miRNA genes are located at fragile chromosomal sites and in proximity to regions of deletion or amplification that are altered in human cancer, implicating a direct involvement for miRNAs in tumorigenesis (8). Families of miRNAs that share similar “seed” sequences or are located in close proximity on a single genomic locus may be co-expressed to form miRNA regulatory networks in particular physiological or pathological contexts. miRNAs that are underexpressed in cancers are tumor suppressors (loss of miRNA contributes to the malignant phenotype), while highly expressed miRNAs function as oncogenes (gain of miRNA contributes to the malignant phenotype). These expression changes control many genes involved in cell proliferation or apoptosis. Therefore, expression profiles of miRNAs may be applied as biomarkers for cancer diagnosis.

The first study on miRNA expression in OS, published by Gao et al. (9), identified 182 miRNAs from a human OS cell line, indicating that miRNAs may contribute to the pathogenesis of OS. Recently, whole genome analysis of DNA copy number, mRNA gene expression, and miRNA transcript profiling performed in seven OS patient tumors identified a signature of 38 differentially expressed miRNAs in OS tumors compared to normal osteoblasts (10). Of these, expression levels of 28 miRNAs were downregulated and 10 were upregulated ≥10-fold in tumors versus osteoblasts, providing likely candidates for further investigation. Other miRNA profiling studies have shown altered expression of several distinct miRNAs in OS tumors including miR-135b, miR-150, miR-542-5p, and miR-652 that were validated in a separate group of tumors (11). The analysis of common insertion site (CIS)-associated genes identified three miRNAs (miR-181, miR-17-5p, and miR-26a-5p) as significant upstream regulators in human OS (12). Also, aberrant expression of individual miRNAs is well-recognized to play a role in the initiation and progression of various cancers. The oncogenic miR-17–92 cluster is overexpressed in several types of cancer and promotes cell proliferation (13). In contrast, downregulation of the miR-15/16 family increases expression of anti-apoptotic proteins B-cell lymphoma 2 (Bcl-2) and myeloid leukemia cell differentiation protein (Mcl-1) (14) and loss of let-7a facilitates amplification of the c-myelocytomatosis virus (*MYC*) oncogene to promote B-cell tumorigenesis (15). Characterization of these miRNAs that display altered expression in OS may provide distinct miRNAs or miRNA signatures related to particular molecular patterns associated with this disease.

Unlike many other types of cancer, there are no traditional biomarkers for OS. The presence of metastatic disease and histologic response assessed following adjuvant chemotherapy (i.e., the extent of necrosis) are the only generally accepted predictors of event-free survival (16). The identification of new diagnostic miRNA biomarkers has the potential to complement existing risk prediction models and could eventually have a prognostic value in this disease. miRNA expression signatures are undergoing clinical investigation in pediatric patients with central nervous system (CNS) tumors (NCT01595126, NCT01556178), CNS tumors along with leukemia and lymphoma (NCT01541800), acute myeloid leukemia (AML) (NCT01229124), and neurofibromatosis Type 1 (NF-1) (NCT01595139). Also, the molecular analysis of solid tumors (MAST) clinical trial (NCT01050296) is designed to prospectively characterize the molecular, cellular, and genetic properties of primary and metastatic solid tumors in patients including OS. These studies present a novel opportunity to investigate the expression of miRNAs in the blood, body fluids, and tissue of patients as an early predictor of cancer as well as a marker of response to therapy. Of note, one Phase 1 clinical trial conducted by MiRNA Therapeutics Inc. is evaluating miR-34 as an miRNA replacement therapy in patients with non-resectable primary liver cancer, with liver metastasis from other cancers, and a cohort of patients with hematological malignancies (NCT01829971).

The various genomic and molecular alterations, which are linked to the development and progression of OS are well established. These include germline mutations, gene amplifications and deletions, overexpression and activation of receptor tyrosine kinases (RTKs), enhanced cell proliferation, resistance to apoptosis, metastasis, drug resistance genes, and miRNAs [reviewed in Ref. (17); and available at http://osteosarcoma-db.uni-muenster.de]. These alterations mediate changes that affect the expression and function of several genes and gene regulatory networks. miRNA profiling and computational analyses have identified associations between miRNAs and many gene and gene products linked to these aberrant factors. This review discusses some of the prominent pathological factors of OS that may be regulated by miRNAs and highlights miRNAs that are validated in preclinical OS models.

## miRNAs in the Pathogenesis of OS

### Germline mutations

Osteosarcoma is characterized by complex, unbalanced karyotypes, and the pattern of abnormalities varies among patients. Numerical and structural chromosome abnormalities are detected in the majority of OS tumors (58%) (3, 17–19). Common numerical chromosomal abnormalities are polyploidy, caused by errors in mitosis, aneuploidy, germline mutations, deletions, duplications, and unbalanced translocations. These include gain of chromosome 1, loss of chromosomes 9, 10, 13, and/or 17, partial or complete loss of the long arm of chromosome 6 and ring chromosomes (7%) (19, 20). Characteristic reciprocal translocations are absent in OS and rearrangements of chromosomes 11, 19, and 20 are frequent structural abnormalities (21). Two of the most prominent genes that harbor germline mutations are the retinoblastoma tumor suppressor gene (*RB1*) and the *TP53* tumor suppressor gene (2). These genes are important for mitotic checkpoints and are thought to be the underlying cause of chromosomal instabilities. Most OS tumors contain inactivation of both the retinoblastoma (Rb) and p53 pathways.

#### *RB1* 

The retinoblastoma protein (pRb) was the first described tumor suppressor. pRb1 is a checkpoint protein that binds the E2F family of transcription factors and inhibits cell-cycle progression. The activation of cyclin-dependent kinases (CDK) and cyclins by mitogenic signals phosphorylates pRb, which dissociates from E2F transcription factors. This leads to the activation of E2F target genes to facilitate the G1/S transition and S-phase progression (22). Thus, loss of function of the *RB1* gene drives tumorigenesis in many adult and pediatric cancers including OS (23, 24), retinoblastoma (25), medulloblastoma, supratentorial primitive neuroectodermal tumor (sPNET) (26), and acute lymphoblastic leukemia (ALL) (27). Gene mutations occur in 20–40% of patients with sporadic OS (28) and loss of heterozygosity (LOH) at 13q, the site of location of the *RB1* gene occurs in approximately 70% of OS cases (29). Germline mutations along with genetic loss or deletions of *RB1* are associated with inactivation of pRb. These are considered as high risk factors for the development of OS and are linked to poor outcome (29).

An miRNA signature consisting of miR-9-5p, miR-138, and miR-214 was predicted to target mRNA genes that encode proteins involved in pRb-signaling in OS (30). However, the experimental validation of these miRNA:mRNA interactions has not been conducted. Other miRNAs including miR-449a, miR-449b, and the miR-17–92 locus have been described in the regulation of Rb/E2F (E2F transcription factor) pathway in many cancers (31). Mechanistically, miR-449a and miR-449b were direct transcriptional targets of the E2F transcription factor 1 (E2F1) and negative modulators of pRb phosphorylation by inhibition of (cyclin-dependent kinase 6) *CDK6* and cell division cycle 25A (*CDC25A*) (32). This was consistent with the previously identified regulation of CDK/pRb/E2F1 through an auto-regulatory feedback mechanism by miR-449a (33). Interestingly, miR-449a was expressed in low levels in OS cell lines and tumors and directly targeted the binding site within the 3′-UTR of the *BCL2* mRNA (34). In a separate study, restoration of miR-29a in osteoblastic cells using miRNA mimetics was shown to repress *BCL2* mRNA and induce E2F3 and its transcriptional target, E2F1 (35). Collectively, these findings support a pro-apoptotic and tumor suppressor role for miR-29a that may participate with miR-449 to regulate the Rb/E2F signaling network in OS.

miR-17–92 is a well-studied polycistronic miRNA cluster in cancer. The miR-17–92 locus contains 15 miRNAs that form four “seed” families, miR-17, miR-18, miR-19, and miR-92. Many of these are reported to be amplified in diffuse B-cell lymphoma, lung, breast, and pancreatic cancers, as well as OS (13, 36, 37). Direct target genes of miR-17–92 include *E2F1*, phosphatase and tensin homolog (*PTEN*), and *p21* (13). Two members of the miR-17–92 cluster (miR-17-5p and miR-18a) were among five highly expressed oncogenic miRNAs in several OS cell lines that were suggested to be predictive of poor disease prognosis (36). In the Sleeping Beauty (SB) transposon-based forward genetic screen, miR-17-5p was one of the three enriched upstream regulators identified in an OS mouse model (12). Future studies to establish the function of miR-17–92 in OS are warranted.

#### *TP53* 

The human p53 gene encodes the p53 tumor suppressor protein that plays a crucial role in maintaining genomic stability. The p53 tumor suppressor functions as a transcription factor that regulates the expression of various genes that are involved in cell-cycle arrest, DNA repair, and apoptosis. In normal, unstressed cells, mouse double minute 2 (MDM2) binds and inhibits p53 function to allow p53 degradation via the ubiquitin/proteasome pathway. In stressed cells, p53 is stabilized, and p53 transcriptional activity is promoted. Alterations in the *TP53* gene are associated with functional inactivation of p53 and less favorable prognosis in OS (38). Genetic abnormalities of p53, such as allelic loss (75–80%), gene rearrangements (10–20%), or point mutations (20–30%) are reported in 50% of OS patients (39). The *MDM* gene is also amplified in 16% of OS patients and is associated with aggressive disease (40). Patients with the Li–Fraumeni syndrome with a germline mutation of *TP53* are predisposed to developing OS.

Studies involving various cancer types have validated several miRNAs that are components of the signaling cascades that regulate p53 expression. miR-125b, miR-504, miR-25, and miR-30d are reported to directly bind the p53 mRNA and negatively regulate p53 expression (41). In addition, miR-34a, miR-192, miR-194, miR-215, miR-605, and miR-29 regulate upstream components and indirectly activate p53 (41). Of these, miR-34a is a key tumor suppressor that regulates numerous genes that are involved in DNA damage and repair. A positive feedback loop mediated by p53 target sites in the miR-34a promoter also controls transactivation of miR-34a by p53 (42, 43). Mutations in *TP53*, functional inhibition of p53, and hypermethylation of the miR-34a promoter are all associated with the loss of miR-34a expression in tumors (44). In a p53-expressing OS cell line, DNA damage-induced miR-34a expression was dependent on p53, which in turn led to the induction of cell-cycle arrest, promotion of apoptosis, and DNA repair (45). This was not observed with p53-deficient OS cells, illustrating that miR-34a was a downstream target of p53. Restoration of miR-34a with miRNA mimics in OS cells repressed p53 and runt-related transcription factor 2 (RUNX2) and suppressed tumor cell growth (46). Notably, miR-34a was demonstrated to be a target of C/EBPα CCAAT/enhancer-binding protein-alpha (C/EBPα, encoded by the gene *CEBPA*) during granulopoiesis, and low expression in leukemic blasts with CEBPA mutations elevated levels of E2F3 and its transcriptional target, E2F1 (47). A clinical study of the role of miR-34a and miR-194 in pediatric patients with AML with mutated CEBPA was recently concluded by the Children’s Oncology Group and the results of this study are pending (NCT01057199).

p53 can transcriptionally induce miR-192 and miR-215 in several types of cancer (48). These miRNAs were markedly downregulated in OS (49), and transactivation of miR-192 and miR-215 by p53 was also reported in OS cell lines (48). The ratio of expression levels of miR-192 and miR-215 has been proposed to differentiate p53-negative and p53-expressing OS patient tumors (49). In addition, miR-215 has been implicated in p53-mediated chemoresistance to methotrexate and the thymidaylate synthase inhibitor, Tomudex, in OS cell lines. Resistance was mediated through repression of the denticleless protein homolog (DTL), a cell-cycle-regulated nuclear, and centrosome protein (50). Together, these findings support miR-34a, miR-192, and miR-215 as candidates for novel biomarkers of prognosis and drug response in OS.

### Gene amplifications

Several genetic deletions and amplification are considered predisposing conditions to OS. Genomic amplifications (homogeneously staining regions) occur in approximately 30% of OS cases (20), which are associated with the action of oncogenes, such as apurinic/apyrimidinic exonuclease 1 (*APEX1*), cellular homolog of avian *MYC*, RecQ protein-like 4 (*RECQL4*), *CDK4*, *MDM2*, *RUNX2*, and vascular endothelial growth factor A (*VEGFA*) (51). Many of these amplified genes are involved in cellular proliferation, survival, and angiogenesis of OS. Amplification of the 12q13 chromosomal region (containing *MDM2* and *CDK4*) or *INK4A* deletion at location 9p21 can affect both the p53 and pRb pathways, and may sometimes occur simultaneously with *RB* or *TP53* alterations (14). Of these amplified genes, the miRNAs targeting *MYC* and *RUNX2* have been well-characterized in OS.

#### *MYC* 

The *MYC* oncogene encodes a transcription factor that regulates genes that control cell growth and cell-cycle progression (52). Genetic and epigenetic alterations associated with constitutive c-Myc activation promote oncogenesis in a variety of cancers (52). The *MYC* locus is amplified in ~30% of OS tumors (53), and c-Myc protein is overexpressed in the majority of OS cases (54). Thus, dysregulation of c-Myc is an important component of OS pathogenesis. The c-Myc transcription factor globally silences several miRNAs either by inhibition of tumor suppressor miRNAs (including miR-15a/16-1, miR-34 family, miR-23), or activation of oncogenic miRNAs (e.g., miR-17–92). In addition c-Myc forms a feedback regulatory loop involving direct or indirect repression of let-7, a well-recognized tumor suppressor miRNA, through the RNA-binding protein, LIN28 (15, 55). Thayanithy et al. (56) demonstrated significant decreases in expression levels of miRNAs at the 14q32 locus (miR-369-3p, miR-544, miR-134, and miR-382) in OS cell lines and tumors compared to normal bone tissues. This correlated with c-Myc overexpression and enrichment of the miR-17–92 cluster. In addition, miR-135b (57) and miR-33b (58) were demonstrated to directly repress c-Myc in OS cells and restoration inhibited cell proliferation, migration, and invasion. Interestingly, the expression of let-7 family members was attenuated in OS cell lines (59, 60). This was predicted to affect the regulation of oncogenes that influence cell-cycle progression and apoptosis. Since let-7 targets multiple oncogenes including *MYC, RAS, CCND, BCL2* (61), this miRNA may be an interesting candidate for future investigation in this disease.

#### *RUNX2* 

The *RUNX2* gene located on chromosome 6p12–p21 is frequently amplified in OS and is associated with tumor growth (20). This gene encodes a transcription factor that is necessary for both osteoblast differentiation and chondrocyte maturation. RUNX2 is linked to many human cancers including breast, prostate, and bone cancer and also cancer metastasis in bone (62). High expression levels often correlate with poor response to chemotherapy (63, 64). The RUNX2 protein has been shown to directly interact with p53 and pRb transcription factors (58), but the precise function of RUNX2 in OS pathogenesis is unclear. An inverse correlation of expression between miR-23a and *RUNX2* mRNA levels in OS cells and tumors was demonstrated by He et al. (65). An association between miR-23a and *RUNX2* and chemokine (C–X–C motif) ligand 12 (*CXCL12*) mRNA was demonstrated as enrichment of miR-23a suppressed transcriptional activity of RUNX2 and inhibited proliferation in OS cells and xenograft tumors (65). These studies indicate a tumor suppressor function for miR-23a in this disease. In addition, miR-103a was reported to play a role in the regulation of osteoblast differentiation by directly targeting the 3′-UTR of *RUNX2* mRNA to inhibit matrix mineralization and bone formation (66). Other miRNAs, miR-135 and miR-203, were identified to modulate RUNX2 in breast cancer cells (67). Inhibition of RUNX2 interacting proteins by miRNAs also affected RUNX2 stability and transactivation potential. Protein expression levels of RUNX2 and the co-transcription factor, SATB2, were regulated by miR-205 and overexpression of the special AT-rich sequence-binding protein 2 (SATB2) activated RUNX2 and reversed the inhibitory effects of miR-205 on osteoblastic differentiation (68). These studies provide more comprehensive details on the involvement of miRNAs involved in osteoblast regulation and OS.

### Receptor tyrosine kinase activation

Aberrant activation of RTKs and their ligands promote malignant progression in OS. These RTKs include epidermal growth factor receptor (EGFR), insulin-like growth factor 1 receptor (IGF-1R), vascular endothelial cell growth factor receptor (VEGFR), platelet-derived growth factor receptor (PDGFR), and mesenchymal–epithelial transition factor (c-Met). RTK activation via gene mutations, gene amplifications, protein overexpression, and/or ligand-dependent autocrine/paracrine loops has been demonstrated in patient primary tumors, cell lines, and xenograft tumors. This is generally associated with cell proliferation, cell survival and metastasis, and overall poor prognosis. In the past decade, several monoclonal antibodies and small molecule inhibitors targeted against RTKs have been evaluated in pediatric patients with solid tumors including OS (69). These agents were well-tolerated but showed limited single-agent activity in patients. Moreover, resistance to these therapies due to cross-talk between receptors resulted in the activation of compensatory RTK cell survival signaling to facilitate tumor progression (15). A more detailed understanding of biological mechanisms of drug response and resistance will assist in addressing the challenges of RTK inhibition. Recent experimental studies have investigated the role of several tumor suppressor miRNAs in the regulation of *IGF-1R* and *MET* in OS.

#### IGF-1R

IGF-1R is a transmembrane receptor that is activated by IGF-1 and IGF-2 ligands and mediates signaling involved in processes, such as cell proliferation, migration, and differentiation (70). High expression levels of IGF-1R, IGF-1, and IGF-2 have been demonstrated in many cancers including breast, prostate, colon, and pediatric cancer (71, 72). IGF-1R is overexpressed in ~45% of OS patients (72). In 2013, Chen et al. demonstrated that miR-16 expression levels were low in OS cell lines and inversely correlated with *IGF-1R* mRNA levels. miR-16 is a member of the mir-15/16 family that functions as a tumor suppressor in a variety of cancers. These miRNAs target *BCL2* and numerous genes involved in the G1/S transition, such as cyclin D1 (*CCND1*), cyclin D3 (*CCND3*), cyclin E1 (*CCNE1*), and *CDK6* (14). The restoration of miR-16 in OS cells suppressed proliferation by inhibition of IGF-1R and the Ras/Raf/mitogen-activated protein kinase (MAPK) pathway. These findings were significant since MAPK activation is associated with the induction of proliferative and anti-apoptotic signaling in OS (73) and in resistance mechanisms to targeted therapies (74). Han et al. (75) demonstrated that miR-194 directly targeted *CDH2* and *IGF-1R* mRNA to suppress OS cell proliferation and metastasis *in vitro* and *in vivo*. A tumor suppressor role of miR-194 was also described for gastric cancer (76) and lung cancer (77), though bone morphogenetic protein 1 (*BMP1*) and the cyclin-dependent kinase inhibitor *p27(kip1)* were the targets of miR-194 in these cancers. Furthermore, expression of miR-133b correlated negatively with IGF-1R, Bcl2-like 2 (Bcl2L2), Mcl-1, and c-Met protein levels in OS cells (78). This suggests the potential of miR-133b to function as a master regulator of critical genes, which control cell survival in OS. These insights into the miRNA-mediated regulation of IGF-1R provide new details of biological mechanisms of response and resistance to IGF-1R inhibition in preclinical models of OS.

#### MET

The *MET* oncogene encodes the receptor for the hepatocyte growth factor (HGF), a cytokine that stimulates invasive growth of normal and neoplastic cells. The c-Met receptor is overexpressed in a variety of human malignancies, including sarcomas, and particularly in chordoma (94.4%), chondrosarcoma (54.2%), and OS (23.3%), determined in 122 cases of malignant bone tumors (79). Activation of c-Met increases phosphatidylinositol-3-kinase (PI3K)/Akt, Src, c-Jun N-terminal kinase, signal transducer and activator of transcription 3 (STAT3) and MAPK pathway signaling, and is implicated in acquired resistance to EGFR and angiogenesis inhibitors (80, 81). Two miRNAs that were found to directly target *MET* mRNA in OS were miR-34a (82) and miR-199a-3p (83). This was consistent with previous reports that c-Met is one of the common targets for the miR-34 family (42). The overexpression of c-Met in tumors of p53-deficient mice and in Li–Fraumeni patients (84) suggests that miR-34/p53/c-Met may form a gene regulatory network that cooperatively controls tumor progression in OS. Restoration of either miR-34a or miR-199a-3p with respective miRNA mimics in OS cell lines achieved a reduction of cell migration and invasion. In addition, the repression of miR-199a-3p was associated with inhibition of mechanistic target of rapamycin (mTOR) as well as STAT3. In an independent investigation, miR-199a-3p and let-7a were evaluated in OS cells using lipid-modified dextran-based polymeric nanoparticles as a delivery system (85). Studies of effective delivery methods for miRNAs are relevant to achieve optimal miRNA enrichment or gene silencing in OS cells and tumors. These studies demonstrated that a lipid-modified dextran-based polymeric nanoparticle platform may be an effective non-viral carrier for efficient and effective miRNA delivery *in vivo*.

### Cell proliferation

The PI3K/Akt and MAPK pathways are two of the most frequently activated signal transduction pathways associated with OS. They contribute to disease initiation and development, uncontrolled cell proliferation, tumor cell invasion and metastasis, cell-cycle progression, inhibition of apoptosis, angiogenesis, and chemoresistance. The PI3K/Akt pathway is activated by the binding of ligands to respective RTKs (including IGF-1R, c-Met, and EGFR). Downstream signals activate targets involved in cell survival and inactivate pro-apoptotic proteins. The MAPK pathway is also activated by IGF-1R and EGFR signals, which may lead to cross-talk with the PI3K/Akt pathway. Aberrant activation of the MAPK pathway is often linked to lung metastasis (86) and drug resistance in OS (74). The influence of miRNAs on components of these pathways has been studied in OS pathology.

#### PI3K/Akt

The PTEN protein functions as a tumor suppressor that negatively regulates activation of the Akt pathway to inhibit cell proliferation (87). Gene deletions at the *PTEN* locus account for loss of PTEN function in 15–33% of OS patients (88). Consequently, loss of PTEN is associated with tumor progression. *PTEN* mRNA was directly targeted by miR-221 in OS cell lines and high miR-221 levels were shown to correlate with low *PTEN* mRNA and protein expression (89). Enrichment with miR-221 mimics induced OS cell survival while attenuation with antimiRs induced apoptosis, demonstrating that this is an oncogenic miRNA in OS. Notably, inhibition of miR-221 enhanced sensitivity to cisplatin (89), indicating the involvement of miR-221 in drug resistance mechanisms. Other miRNAs including miR-92a and members of the miR-17 and miR-130/301 families were found to show an inverse correlation in expression levels with *PTEN* mRNA expression levels in OS tumors (37) to provide additional candidates for investigation. Of these, inhibition of miR-17 resulted in increased *PTEN* mRNA in OS, which was associated with suppression of tumor growth and metastasis (90).

Another critical component in the PI3K/Akt pathway is the mTOR protein kinase. The mTOR complex consists of mTOR complex-1 (mTORC1), which regulates cellular proliferation, and mTOR complex-2 (mTORC2), which phosphorylates and activates Akt. Direct repression of mTOR by the miR-101 tumor suppressor was reported in OS (91), and inhibition of cell proliferation and apoptosis were mediated through suppression of mTOR. Growth inhibition by miR-101 was also shown in hepatocellular carcinoma cells (92), and miR-101 was also found to contribute to cisplatin-induced apoptosis. Thus, these miR-221/miR-17/PTEN and miR-101/mTOR interactions provide new insight into the role of miRNAs as potential regulators of aberrant PI3K/Akt pathway signaling in OS. Further, the identification of miRNAs associated with resistance to cisplatin is promising for the development of biomarkers of chemoresistance in this disease.

#### MAPK

Epidermal growth factor receptor signaling with activation of the MAPK pathway occurs in 49% of OS cases and is linked to metastatic disease (93). Ras/Raf is upstream of MAPK/ERK kinase (MEK). Aberrant activation of the Ras/Raf/MAPK pathway is known to be specifically associated with OS initiation, progression, and outcome. A direct interaction between miR-217 and KRas was recently reported to participate in MAPK activation in OS (94). In a study evaluating miR-143 in OS tumors, a direct association of elevated EGFR phosphorylation and matrix metalloprotease-9 (MMP-9) levels and low miR-143 expression was reported (95). The MMP family of proteolytic enzymes facilitates tumor cell invasion and metastasis through degradation of various components of the extracellular matrix (96). MMP-9 degrades collagen type IV, the major component of the basement membrane and overexpression is associated with tumor metastasis (96). In this study, EGF promoted activation of EGFR and induced MMP-9 to enhance the ability of OS cells to metastasize. Significantly, miR-143 was reported to inhibit EGFR signaling-dependent OS cell invasion.

### Apoptosis

Apoptosis is a critical event in embryonic development and in maintenance of tissue homeostasis of multicellular organisms. The activation of anti-apoptotic signals facilitates uncontrolled cell growth in cancer cells. The major apoptotic pathways include both the intrinsic and extrinsic pathways. The intrinsic pathway is mediated by mitochondrial components and triggered by intracellular stimuli, such as DNA damage, cytotoxic agents, growth factor suppression, and/or oxidative stress. The extrinsic pathway is initiated by the binding of death ligands, Fas ligand (FasL), tumor necrosis factor (TNF), TNF-related apoptosis-inducing ligand (TRAIL), and TNF-like weak inducer of apoptosis (TWEAK) to the TNF receptor (TNFR) superfamily of death receptors (97). Other key proteins in tumor cells that control apoptosis include the tumor suppressor p53 and PI3K/Akt activation that regulate downstream substrates that trigger or prevent apoptosis, respectively (97). miRNAs directly targeting components of the basic apoptotic pathways in OS have been identified.

#### Intrinsic Apoptotic Pathway

Low levels of miR-133a in OS cell lines and tissues were found to correlate with tumor progression and poor prognosis (98). Restoration of miR-133a inhibited cell proliferation and induced apoptosis in OS cell lines. A similar tumor suppressor role for miR-133a was reported in colorectal cancer (99). The regulatory mechanism of miR-133a involved direct targeting and suppression of B-cell lymphoma-extra large (Bcl-xL) and Mcl-1 proteins. Both Bcl-xL and Mcl-1 are anti-apoptotic proteins that are highly expressed in OS and promote cell survival (100). The miR-29 family comprises three isoforms arranged in two clusters, miR-29b-1/miR-29a located on chromosome 7 and miR-29b-2/miR-29c on chromosome 1, and these miRNAs have been identified as tumor suppressors in chronic lymphatic leukemia (CLL), AML, lung cancer, and breast cancer (101). Low levels of miR-29a were observed in OS, which when restored, induced apoptosis leading to the silencing of *BCL2* and *MCL1* and enrichment of the tumor suppressors *E2F1* and *E2F3* (35). miR-29 is currently being clinically evaluated as a biomarker for primary measure outcome of histone deacetylase inhibitor, AR-42, in adult and pediatric AML patients (NCT01798901). A clinical trial to evaluate the molecular mechanism and clinical significance of the interaction between Twist1 and other epithelial-to-mesenchymal regulators through the miR-29 family is also underway (NCT01927354). Another study investigating the role of miR-29b in patients with oral squamous cell carcinoma has been proposed (NCT02009852). Collectively, these data support a tumor suppressor function of miR-29 and suggest that the use of synthetic miR-29 oligonucleotides or agents increasing miR-29 expression can be incorporated in the study of expression changes of critical genes in OS.

#### Extrinsic Apoptotic Pathway

Fas ligand (FasL or CD95L) is a type-II transmembrane protein that belongs to the TNF family (97). FasL binds the Fas receptor (Fas, also called apoptosis antigen 1, Apo1, or cluster of differentiation 95, CD95) and induces apoptosis. Low Fas expression in OS tumor cells was associated with disease development and progression. Huang et al. demonstrated that miR-20a encoded by the miR-17–92 cluster attenuated *FAS* levels and regulated Fas-mediated apoptosis in OS cells (102). Another miRNA, miR-106a, which was downregulated in OS cell lines and tumors, was associated with regulation of *FAS* (103). miR-106a is part of a miRNA cluster (miR-17, miR-18a, miR-92a, and miR-106b), and cross-talk between miRNAs was suggested to mediate *FAS* repression. Interestingly, the pro-apoptotic gene BH3-only (*BIM*) was suppressed by miR-17 only (103) to support the regulation of Fas-mediated apoptosis by this miRNA cluster in OS cells.

### Metastasis

In general, OS patients with lung metastasis have a poor prognosis. The overall survival rate is low (~25%) for those patients who present with metastases (~20% of all cases) (104). The process of metastasis involves dissemination of cells from the primary tumor, invasion of the extracellular matrix, and proliferation of cells at distant sites (105). A number of factors, including Rho-associated coiled-coil kinase 1 (ROCK1), MMPs, and c-Fos (the cellular homolog of v-fos), are involved in tumor metastasis. Recent studies have identified several miRNAs that directly target the mRNAs encoding these proteins. The identification of miRNAs associated with metastatic disease holds promise as circulating miRNA biomarkers assessing disease characteristics that may be detected in serum and plasma of patients.

#### Rho-Associated Coiled-Coil Kinase 1

The ROCK1 protein serine/threonine kinase is a downstream effector of the small GTPase RhoA, and is a regulator of the actomyosin cytoskeleton. The RhoA/ROCK pathway participates in the process of tumorigenesis in numerous types of cancer. ROCK1 promotes contractile force generation and is involved in cell motility, metastasis, and angiogenesis in cancer cells (106). Several miRNA:*ROCK1* mRNA associations have been described. Generally, *ROCK1*-associated miRNAs, miR-340 (107), miR-335 (108), miR-145 (109), and miR-144 (110), were weakly expressed in OS cell lines and tissues and correlated inversely to ROCK1 overexpression. In addition, low expression levels of these miRNAs were associated with OS progression and metastasis through mechanisms involving ROCK1 to provide initial evidence that supports these miRNAs as predictors of poor prognosis in OS.

#### MMP-13

Matrix metalloproteases are produced either by tumor cells or stromal cells. Overexpression of MMP proteins is an important predictive factor for relapse or nodal metastasis of many carcinomas (111, 112). MMP-13 expression is common in lung metastasis (31). Recent evidence shows the emerging roles of miRNAs in direct repression of MMP through inhibition of gene transcription or (113) inhibition of *MMP* RNA levels (114). Osaki et al. showed that the expression of miR-143 was significantly downregulated in comparison to expression levels in parental (HOS) cell line and subclone (143B) human OS cell line, which shows lung metastasis in a mouse model (31). This finding correlated with MMP-13 upregulation, which implicated MMP-13 as a downstream mediator of miR-143 function in OS metastasis.

#### FOS

FBJ murine osteosarcoma viral oncogene homolog (*FOS*) is the transforming gene identified originally in the FBi and FBR murine sarcoma viruses (115). The c-Fos protein is part of a heterodimeric complex with JUN and is a major component of the Activator Protein-1 (AP-1) transcription factor complex. AP-1 regulates cell growth, differentiation, transformation, and bone metabolism (116). c-Fos is overexpressed in 61% of OS tumors compared to benign and normal tissue (117). It is also enriched in high-grade lesions and in patients with metastases (42%). c-Fos overexpression in transgenic mice was associated with OS development, suggesting a potential role in tumor initiation (118). The correlation between low expression of miR-181b/miR-21 signaling and *FOS* upregulation was made in malignant gliomas (119). miR-181b modulated *FOS* expression by directly targeting the binding site within the 3′-UTR. mir-221 was a predicted gene target of FOS (10), but the integrated analyses of *FOS* mRNA and regulatory miRNAs have not been experimentally studied in OS.

### Drug-resistant genes

Drug resistance is often mediated through the activation of several molecular pathways that inhibit apoptosis and promote cell survival, to compensate for the effects of chemotherapy and targeted inhibition. In addition, increased DNA damage repair and ejection of the drug from the cell by drug efflux pumps reduce the efficacy of many cytotoxic agents. Experimental studies have demonstrated that the altered expression of specific miRNAs that regulate these cellular processes leads to drug resistance in different cancers. In OS cells, overexpression of miR-221 and miR-101 caused cisplatin resistance, mediated through the PTEN/Akt pathway (89, 92), while conversely, increased miR-217 expression levels were associated with reduction in KRas and enhanced sensitivity to quercetin and/or cisplatin (50). In the study conducted by Song et al., miR-215 was shown to induce G2 arrest in OS and colon cancer cells leading to chemoresistance to methotrexate and Tomudex (50).

#### MDR1

An underlying cause of multi-drug resistance (MDR) in OS is the overexpression of one or more of the ATP-binding cassette (ABC) transporters. Many cytotoxic agents are substrates for the *MDR1* (*ABCB1*) gene, resulting in overexpression of P-glycoprotein (P-gp), a 170–190 kDa transmembrane glycoprotein that belongs to the ABC superfamily (120). *MDR* (in particular, *ABCB1*, *ABCG2*, and *ABCC* family members) mediates the efflux of many cytotoxic agents in OS to decrease drug efficacy (120). Zhu et al. (121) have demonstrated that *MDR1*/P-gp expression in human cancer cells was regulated by high levels of miR-27a and miR-451 expression. miR-27a/miR-27a* is a miRNA pair derived from a single precursor. In this study, the sensitivity to and intracellular accumulation of cytotoxic drugs that were transported by P-gp were enhanced by treatment with antagomirs against miR-27a, demonstrating a role in *MDR1*-mediated chemoresistance in OS. miR-27a/miR-27a* was also shown to promote pulmonary OS metastases formation (122) to suggest this miRNA functions as an oncogene in this disease. In contrast, in head and neck squamous cell carcinoma, miR-27a* was demonstrated as a tumor suppressor by targeting the EGFR signaling axis (123) to illustrate fundamental differences in miRNA expression between OS and other types of cancer.

### Challenges and future perspectives

**Figure 1:**
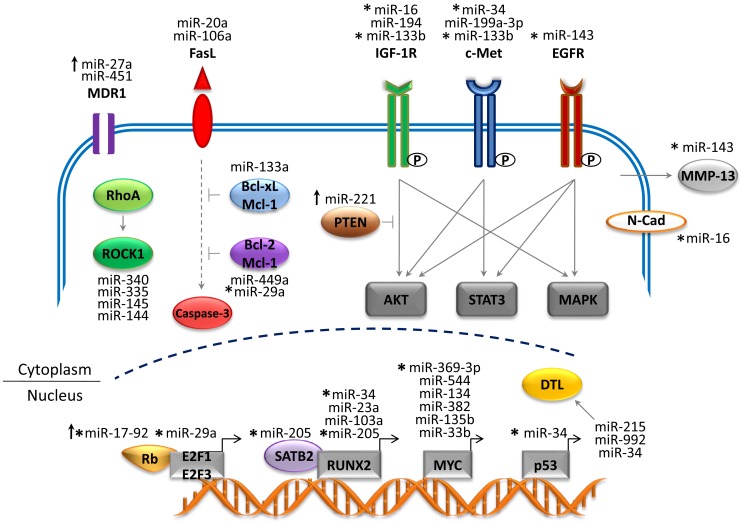
**Network of miRNAs and target genes in OS**. The figure depicts altered miRNA genes that play a critical role in the development and progression of OS. The majority of miRNAs are downregulated (tumor suppressors) and target genes are overexpressed (oncogenes). Upregulated miRNAs (oncogenes) are depicted by upward arrows and target genes are repressed (tumor suppressors). Abbreviations: MDR1, multi-drug resistance 1; FasL, Fas ligand; IGF-1R, insulin-like growth factor 1 receptor; EGFR, epidermal growth factor receptor; ROCK1, Rho-associated coiled-coil kinase 1; Bcl2, B-cell lymphoma-2; Bcl-xL, B-cell lymphoma-extra large; Mcl-1, myeloid leukemia cell differentiation protein; PTEN, phosphatase and tensin homolog; MMP-13, matrix metalloprotease-13; N-Cad, N-Cadherin; SATB2, special AT-rich sequence-binding protein 2; RUNX2, runt-related transcription factor 2; DTL, denticleless protein homolog). Solid gray arrows represent activated signaling pathway; solid blunt lines represent inhibition of signals; dotted gray lines represent indirect signaling pathways.

Recent studies have generated a vast amount of DNA sequencing and genomic data that have provided tremendous insight into the molecular pathology of OS (4). Several genetic and epigenetic alterations in OS have been established that are linked to the development, proliferation, and survival of tumor cells (17, 19). The mapping of human miRNA genes has also identified specific miRNAs in OS that modulate gene expression and cellular processes. This has provided new insight into the complex genetic mechanisms of OS tumorigenesis. Many of these miRNA genes are located in cancer-associated genomic regions or in fragile sites (8, 18) and are reportedly associated with the development, progression, and metastasis of OS tumors (represented in Figure 1 and summarized in Table 1). miRNAs are intriguing molecules, as the expression patterns appear to be tissue and cancer-type specific, and the small size is amenable to development for clinical applications. Of interest, circulating miRNAs from tumor cells that are detected in the blood of patients with cancer present a novel opportunity to use miRNAs as an early predictor of cancer as well as a marker of response to therapy.

**Table 1:** **Prominent clinopathological factors associated with OS and regulatory miRNAs that are validated in preclinical OS models**.

OS-associated factor	Target gene/pathway	miRNA	Altered protein(s)	miRNA function	Reference
Germline mutation	*RB1*	miR-17–92	Not verified in OS	Oncogene	(36, 37)
	*TP53*	miR-34	p53	^a^TS	(45)
		^b^miR-192	p53/RUNX2	TS	(46)
		^b^miR-215	p53	TS	(48)
Gene amplification	*MYC*	^b^miR-369-3p	c-Myc	TS	(56)
		^b^miR-544			
		^b^miR-134			
		^b^miR-382			
		miR-135b	c-Myc	TS	(57)
		miR-33b	c-Myc	TS	(58)
	*RUNX2*	miR-23a	RUNX2/CXCL12	TS	(65)
		miR-205	RUNX2/SATB2		(66)
Receptor tyrosine kinase activation	*IGF-1R*	miR-16	IGF-1R	TS	(124)
	*MET*	miR-194	IGF-1R/N-Cadherin	TS	(75)
		miR-133b	IGF-1R/Bcl2L2/Mcl-1/c-Met	TS	(78)
		miR-34	c-Met	TS	(82)
		miR-199a-3p	c-Met	TS	(83)
Cell proliferation	PI3K/Akt	miR-221	PTEN	Oncogene	(89)
		miR-17	PTEN	Oncogene	(37)
	MAPK	miR-143	pEGFR	TS	(95)
Apoptosis	Intrinsic pathway	miR-133a	Bcl-xL/Mcl-1	TS	(100)
		miR-29	Bcl2/Mcl-1/MMP	TS	(35)
	Extrinsic pathway	miR-20a	FasL	TS	(102)
		miR-106a	FasL	TS	(103)
		miR-17	BIM	TS	(103)
Metastasis	*ROCK1*	miR-340	ROCK1	TS	(107)
		miR-335	ROCK1	TS	(108)
	*MMP-13*	miR-145	ROCK1	TS	(109)
		miR-144	ROCK1	TS	(110)
	*FAS*	miR-143	MMP-13	TS	(31)
		miR-20a	FasL	TS	(102)
Drug resistance	*MDR1*	miR-27a	P-gp	Oncogene	(121)

*^a^TS, tumor suppressor*.

*^b^miRNA signature*.

Research characterizing distinct OS-associated miRNAs is still in its infancy. The dysregulation of miRNAs in OS is likely influenced by a variety of factors, which are only starting to be understood. Since OS is a disease that is marked by genetic abnormalities including mutations, single-nucleotide polymorphisms (SNPs), and gene amplifications, it is expected that these alterations may also affect miRNA function. Mutations in the miRNA recognition sites of target mRNAs may affect miRNA binding, resulting in escape from regulation by a specific miRNA. Gene mutations and sequence variation mutations affecting miRNA sequences can potentially affect either processing and/or expression of mature miRNAs to prevent recognition of mRNA targets. However, at present, there is no clear association between mutations and SNPs identified in miRNA precursors in tumors, and cancer cell lines. These are not generally attributed to tumorigenesis and do not alter the secondary structure or function of the mature miRNAs (125, 126). Only few studies of this type have been conducted in OS specifically. Further screening for genetic variants in miRNA genes warrants investigation to determine whether genetic aberrations in miRNAs are integrated into the known cytogenetic abnormalities observed in OS.

Many of the miRNAs discussed in this review are preliminary findings based on *in vitro* studies using cell lines derived from OS tumors and are not fully validated *in vivo* or in functional studies. The robust confirmation of individual or miRNA signatures in preclinical disease models is important for potential applications to cancer treatment. Reduced levels of mature miRNAs in tumors may be a consequence of true absence (lack of inheritance), secondary loss (genetic loss, epigenetic silencing), or defects in biogenesis pathways or transcriptional repression. Quantitative real-time reverse transcription polymerase chain reaction (qRT-PCR) and oligonucleotide microarray (microchip) analysis are the most common methods for measuring miRNA levels, but there is currently no standardized technique for evaluation of miRNA expression, which is critical for clinical translation. In addition, miRNA potential targets can be predicted using computational algorithms, such as TargetScan (127) and microRNA.org (128). By computer predictions and stable isotope labeling with amino acids in cell culture (SILAC), a single miRNA has multiple targets and is capable of inhibiting the translation of hundreds of proteins (129). These are valuable tools for the integrated and functional analyses of miRNA and mRNA targets, and miRNA gene networks, which are also essential for understanding global miRNA roles in OS tumors.

Importantly, miRNA targets are tissue specific and the regulatory roles are in particular physiological or pathological contexts. Approximately 60% of mRNAs have evolutionarily conserved sequences that are predicted to bind miRNAs (130). Thus, the expression of target genes may be controlled by several different miRNAs, and cross-talk between miRNA networks may affect an individual miRNA-based effect. Consequently, an individual miRNA may have oncogenic or tumor suppressor properties in different cell types. Finally, further research to develop strategies for effective and safe miRNA delivery systems is needed. Localized delivery or the use of polyethylene glycol (PEG) in PEGylated liposomes, lipidoids, and biodegradable polymers are being tested [reviewed in Ref. (131)]. Improvements in delivery formulations will reduce the risk of hepatotoxicity, organ failure, and death in preclinical mouse models (131). The design of miRNA precursor mimics (e.g., short hairpin RNAs) or true pre-miRNAs may also minimize toxic side effects while retaining targeted functions. Nonetheless, these small molecules have greatly enhanced our knowledge of the molecular mechanisms that regulate gene expression in OS, and it is hoped miRNAs will be successfully developed to improve the current management of OS.

## Conflict of Interest Statement

The authors declare that the research was conducted in the absence of any commercial or financial relationships that could be construed as a potential conflict of interest.
